# Bleomycin Electrosclerotherapy (BEST) for treatment of slow-flow vascular malformations in children

**DOI:** 10.1038/s41390-025-04455-6

**Published:** 2025-10-03

**Authors:** Constantin Goldann, Anna Deleu, Marie Sophie Schüngel, Julius H. Loeser, Alena Akinina, Moritz Guntau, Moritz Wildgruber, Vanessa F. Schmidt, Walter A. Wohlgemuth, Richard Brill

**Affiliations:** 1 Universitätsklinikum Halle (Saale), University Clinic and Policlinic for Diagnostic and Interventional Radiology, Halle (Saale), Saxony-Anhalt Germany; 2 Department of Diagnostic and Interventional Radiology and Neuroradiology, München Klinik Harlaching, Munich, Bavaria Germany; 3 Technical University of Munich, Faculty of Medicine, Munich, Bavaria Germany; 4 Department of Diagnostic and Interventional Radiology, University Hospital Würzburg, Wuerzburg, Bavaria Germany; 5 Department of Radiology, LMU University Hospital, Ludwig-Maximilians-Universität München, Munich, Bavaria Germany

## Abstract

**Background:**

This prospective study evaluates the effectiveness and safety of Bleomycin electrosclerotherapy (BEST) for treating slow-flow vascular malformations in a pediatric cohort. While retrospective studies have reported its efficacy, prospective data in pediatric populations are limited.

**Methods:**

30 pediatric patients (mean age: 9.5 years) with venous, veno-lymphatic, or capillary-veno-lymphatic malformations were enrolled in the study between 2020 and 2021 and received at least one BEST treatment. Follow-up continued through 2024 (mean: 25 months). MRI volumetry and clinical evaluation were performed at each follow-up.

**Results:**

A total of 58 sessions (mean: 1.81 per patient) led to a median lesion volume reduction from 232 cm³ (range: 1.69 cm³–9814.12 cm³) pre-treatment to 41 cm³ (range: 0.57 cm^3^–1789.63 cm^3^) after the final session, corresponding to mean relative volume reduction of 59.4%. Symptoms resolved in 2 patients, improved in 18, and remained unchanged in 4. Minor side effects included skin hyperpigmentation (*n* = 6), inflammation (*n* = 3), temporary motion restriction (*n* = 1), and lymphorrhea (*n* = 1). No serious complications were observed.

**Conclusion:**

BEST is a safe, effective treatment for pediatric slow-flow malformations, achieving lesion reduction and symptom relief. Future studies are warranted to optimize treatment protocols and establish long-term benefits.

**Impact Statement:**

- Bleomycin Electrosclerotherapy (BEST) is a safe and effective treatment for slow-flow vascular malformations in pediatric patients, achieving lesion volume reduction and symptom improvement.
- The study assesses BEST exclusively in a pediatric cohort using standardized MRI-based volumetry alongside clinical outcomes.
- BEST can be effective not only in refractory cases but also as a first-line or early-line treatment option, broadening its potential indications.
- By indicating a cumulative therapeutic benefit of repeated BEST sessions, the study encourages further investigations into tailored, session-based treatment strategies for large or progressive malformations.

## Introduction

The International Society for the Study of Vascular Anomalies (ISSVA) classifies vascular malformations as slow-flow or fast-flow based on their hemodynamic characteristics, which influence treatment and prognosis.^1^ Venous and lymphatic malformations (VMs and LMs) are common slow-flow anomalies that typically present in childhood, causing symptoms like pain, swelling, thromboembolic events, and functional or aesthetic impairments.^2^

Sclerotherapy with agents like Bleomycin is the most common minimally invasive treatment of slow-flow malformations. Although primarily known as a cytostatic agent, Bleomycin has been successfully used off-label as a sclerosing agent for years. A new approach, known as Bleomycin Electrosclerotherapy (BEST), has emerged as a promising treatment. This technique builds on the established use of Bleomycin in electrochemotherapy, a method commonly applied to cutaneous tumors and skin metastases.^3^ The principle of BEST involves reversible electroporation, a process that temporarily increases the permeability of cell membranes and blood vessels, potentially enhancing drug delivery to target tissues.^4–6^

Current literature on BEST for slow-flow malformations, though limited, includes valuable insights from case reports and retrospective case series^7–9^ conducted on mixed patient groups encompassing both pediatric and adult populations. Our study exclusively focuses on a pediatric population, employing a prospective design to evaluate the effectiveness and patient outcomes of BEST in treating slow-flow malformations.

## Materials and methods

This prospective observational study was conducted at the tertiary care Interdisciplinary Vascular Anomalies Center at the University Hospital Halle (Saale), Germany. The aim of the study was to evaluate the efficacy and safety of BEST in pediatric patients undergoing treatment for slow-flow malformations.

The study was approved by the Institutional Review Board of the Martin-Luther-University Halle-Wittenberg (approval number 2019-150). Written informed consent was obtained from all patients and/or legal guardians prior to their enrollment in the study.

### Patient selection

A total of 30 pediatric patients (under age 18) were recruited between 2020 and 2021, with follow-up continuing until December 2024 (mean follow-up period of 25 months). Eligibility criteria included undergoing at least one BEST treatment session and having an MRI both before the first treatment and at follow-up. Patients were excluded from the study if they did not attend the follow-up assessment, if follow-up MRI imaging was unavailable or if MRI evaluation was not feasible (e.g., due to artifacts or incomplete protocol). Diagnosis of simple or combined venous malformations was based on patient history, ultrasound, magnetic resonance imaging (MRI), and clinical examination findings. Collected demographic data included age, sex, and details of prior treatments.

### BEST treatment protocol

**Fig. 1:**
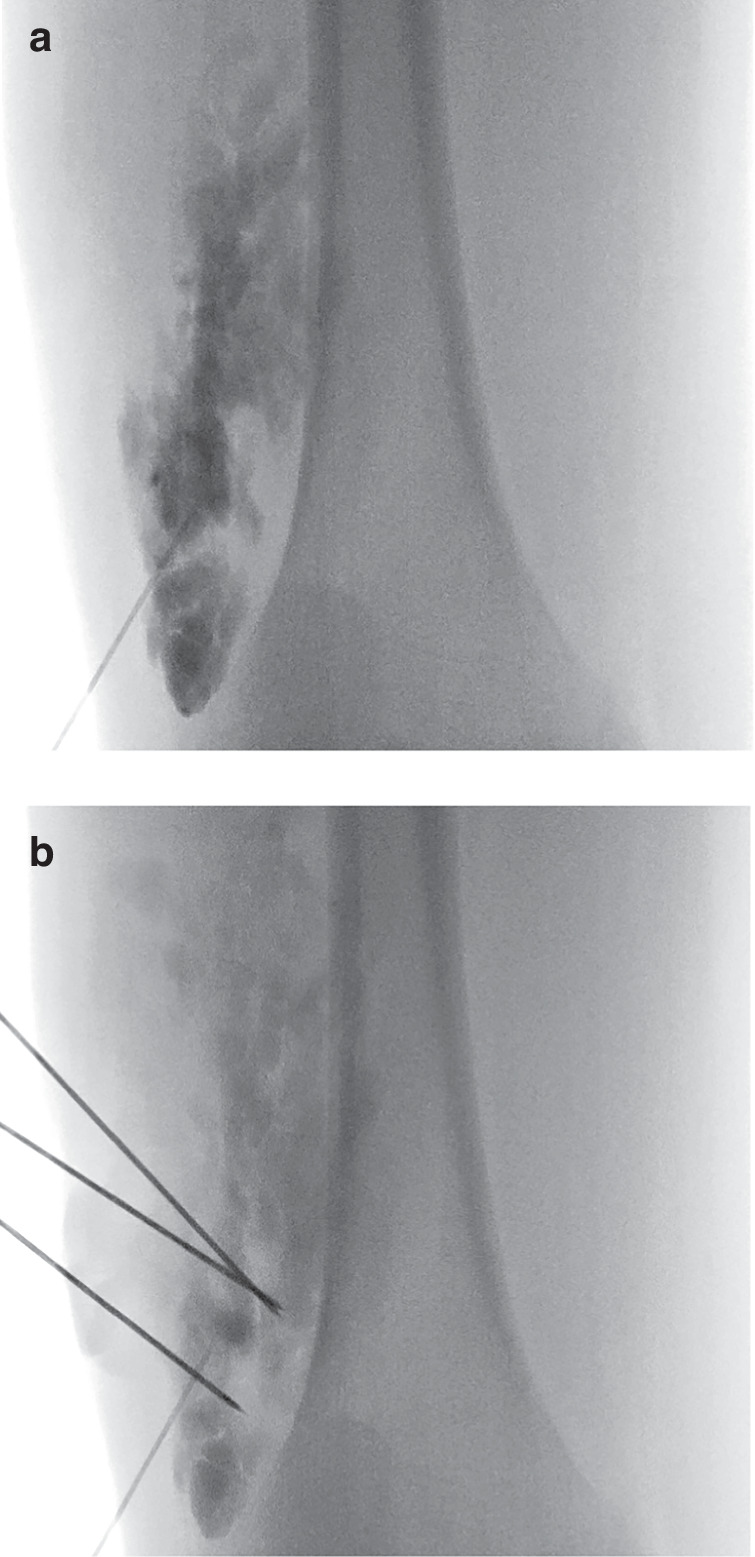
Female patient (13 years) with a venous malformation on her right thigh. **a** Injection of contrast agent into the vascular malformation. **b** Three needle electrodes positioned in the venous malformation under fluoroscopic guidance.

Patients received one or more BEST treatments. Before the intervention, laboratory tests including creatinine, C-reactive protein, complete blood count, liver function tests, D-dimer, and fibrinogen levels were obtained. If the patient’s history or current clinical presentation suggested pulmonary disease, a pulmonology consultation was obtained, and further investigations such as chest X-ray were performed as recommended by the pulmonologist. All interventions were performed under general anesthesia. First, color-coded duplex sonography was performed. In cases of direct application of bleomycin, ultrasound-guided puncture was performed and contrast agent (Imeron 300; Bracco Imaging Deutschland GmbH, Konstanz, Germany) was applied. Correct needle positioning was then confirmed under fluoroscopy to rule out connections to the deep venous system and estimate the required bleomycin volume for intralesional injection based on visualized lesion size (Fig. 1a). For larger and complex malformations, systemic bleomycin application adapted to the bodyweight was also considered.

The following BEST treatment consisted of three main steps: (1) electrode positioning, (2) intravenous or intralesional Bleomycin infusion (or intralesional injection for smaller lesions), and (3) application of short electric pulses for reversible electroporation. Following the latest guidelines for electrochemotherapy,^10^ after an intravenous short infusion, a waiting period of 8 minutes allowed for adequate distribution of Bleomycin in the bloodstream and monitoring for any immediate adverse reactions. If Bleomycin was injected directly into the lesion, the waiting time was reduced to 1–3 min. The maximum bleomycin dose was limited to 1 mg/5 kg body weight per intervention and <5 mg/kg body weight cumulative dose in case of multiple treatment sessions. The maximum Bleomycin concentration was 0.25 mg/ml for both intravenous and intralesional application. Depending on size and location of the malformation, different electrode types were used, including adjustable hexagonal or linear electrodes, finger electrodes, or needle electrodes (Fig. 1b). Electric pulses were applied using an electrical pulse generator (Cliniporator VITAE; IGEA SpA, Carpi, Italy). For each treatment session, the anatomical location of the lesion, the number of electroporation cycles, and the administered Bleomycin dose were documented. Complications were recorded, graded and classified as either temporary or permanent according to the CIRSE classification system.^11^

### MRI-based volumetric assessment

Treatment outcomes were assessed using MRI-based volumetry. To ensure measurement reliability, MRI scans followed a standardized protocol, with measurements performed by a radiologist with five years of experience in vascular malformation imaging and subsequently verified by an independent radiologist with over 20 years of experience. Equatorial and polar diameters of the slow-flow malformations were measured in T2-weighted short-tau inversion recovery (STIR) fat saturated sequences. Volumes were calculated using the rotational ellipsoid formula $V=\left(\frac{{{\rm{\pi }}}}{6}\right)\times {{D}_{{{\rm{e}}}}}^{2}\times {D}_{{{\rm{p}}}}$, where ${D}_{{{\rm{e}}}}$ is the equatorial diameter and ${D}_{{{\rm{p}}}}$ is the polar diameter. The pre-treatment volume served as the baseline for all subsequent measurements. MRI scans were performed at each follow-up visit. Based on these volumetric measurements, absolute and relative volume reductions were calculated. Absolute volume reduction was defined as the difference between the baseline and follow-up volumes. Relative volume reduction was calculated as the percentage decrease relative to the baseline volume to ensure comparability between patients with markedly different lesion sizes.

### Clinical assessment and follow-up

At follow-up (minimum 3 months after the preceding BEST session), patients underwent MR imaging, clinical examination and were questioned regarding changes in their symptoms or complications which occurred during the follow-up period. Clinical response was classified into the following categories: Complete symptom resolution, symptom improvement, no change or symptom worsening/new symptoms. Persistent clinical symptoms were considered an indication for additional BEST sessions with a minimum interval of 3 months between treatments.

## Results

### Patient demographics and baseline characteristics

After exclusion of 6 patients (non-attendance at follow-up), a total of 24 patients were included in the study, with a mean age of 9.5 years (SD 5.1), ranging from 1 to 17 years. The cohort included 10 males and 14 females. The mean follow-up period was 25 months. Prior to study enrollment, 5 patients had not received any interventional treatment. Among the remaining patients, 4 had undergone surgery, 9 had received non-Bleomycin sclerotherapy, 5 had undergone laser treatments, 1 had received compression therapy, and 2 had been treated with Sirolimus. The slow-flow malformations (Table 1) were localized to a single anatomical site in 18 patients, while 6 patients had multiple affected sites.

**Table 1:** Patient characteristics.

Age at start of treatment (median)	9.5 years (range 1–17)
Male : female patients	10 : 14
Slow-flow malformations (total)	24
VM	16
VLM	6
CVLM	2
Previous treatment	
Surgery	6
Laser	3
Sclerotherapy (non-BEST)	9
Compression	4

*VM* venous malformation, *VLM* veno-lymphatic malformation, *CVLM* capillary-veno-lymphatic malformation.

### Treatment characteristics

A total of 58 BEST were performed across all 24 patients, with each patient undergoing an average of 1.81 sessions. Specifically, 12 patients received a second treatment session, 7 underwent a third session, 2 received a fourth session, and 1 patient required a fifth and sixth session.

Depending on the size and anatomical location of the target lesion, linear electrodes were used in 1 session, hexagonal electrodes in 21 sessions, finger electrodes in 24 sessions, and single needle electrodes in 12 sessions. The mean number of application cycles per session was 26.19 (SD 31.11), while the mean Bleomycin dose per session was 3.86 mg (SD 2.61).

### Volumetric outcomes

The median pre-treatment volume of slow-flow malformations was 231.86 cm³. Lesion volumes varied widely among patients, ranging from a 1.69 cm³ venous malformation on the lip of a 1-year-old female, to a 9814.12 cm³ combined venous-lymphatic malformation on the right thigh of a 17-year-old female. Following the first BEST session, the median lesion volume decreased to 73.29 cm³ (range: 1.22 cm^3^–8148.9 cm^3^). After the final treatment session—defined as the last session for patients who underwent multiple treatments or the only session for those who received a single treatment—the median volume further decreased to 40.91 cm³ (range: 0.57 cm^3^–1789.63 cm^3^), resulting in an absolute lesion volume reduction of 82.3%.

**Fig. 2:**
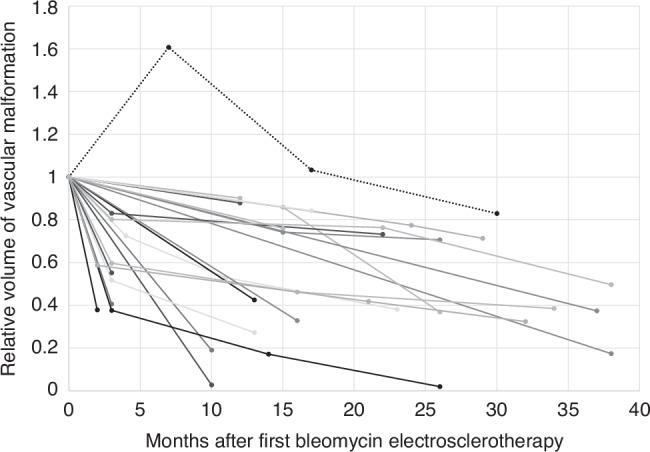
Graph of relative volumes of the vascular malformations per patient over time. One case (marked with a dotted line and square data point) of a patient (age 7 at start of BEST) with a combined vascular malformation showing lesion expansion after the first BEST session but successful volume reduction after the second and third session.

**Fig. 3:**
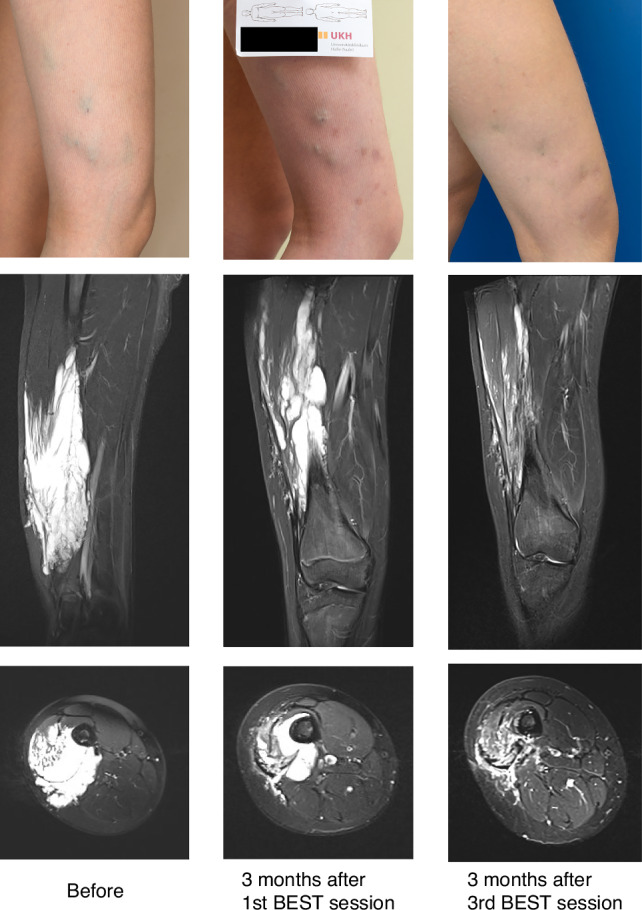
Female patient (13 years at start of therapy) with a venous malformation on her right thigh. Time series over 3 years with photographs and MR images (T2 STIR in coronal and transversal plane) obtained before therapy, 3 months after the 1st BEST session (at age 13) and 3 months after the 3rd BEST session (at age 15).

Across all patients, the median relative volume reduction (Fig. 2) after the first BEST treatment was 38.7%. Following the final treatment session, including patients with both single and multiple treatment sessions, the median relative volume reduction improved to 59.4%. An example case of volume reduction is demonstrated in Fig. 3.

Notably, in one case, a 7-year-old male with a large (before treatment 2157.6 cm^3^) combined veno-lymphatic malformation in the right leg initially experienced an increase in lesion volume after the first BEST session (to 3466.8 cm^3^). However, after subsequent second (2229.7 cm^3^) and third (1789.6 cm^3^) sessions, the malformation was reduced to a volume smaller than its pre-treatment measurement.

### Clinical outcomes

**Fig. 4:**
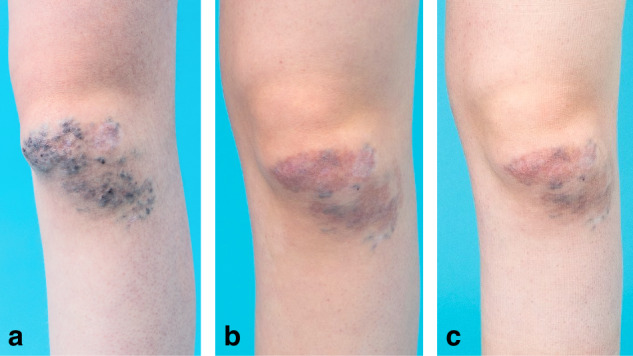
Female patient with venous malformation on her left knee. **a** Before BEST the patient (age 15) had unsuccessful ethanol and polidocanol (ethoxysclerol) sclerotherapies which left discolored scars. **b** 1 year after BEST (age 16) the lesion volume and the blueish superficial veins decreased, while some hyperpigmentation occurred. **c** Two years after BEST (age 17), the hyperpigmentation faded slightly, the superficial veins did not reappear, and the lesion volume remained reduced.

Among the total of 24 patients, changes in symptoms at the final follow-up were as follows: complete resolution of symptoms was observed in 2 patients, improvement in 18 patients, no change in 4 patients. No cases of symptom worsening were observed. Swelling, livid discoloration, redness, thrombophlebitis, superficial dysplastic veins, sensory abnormalities, and functional limitations showed overall improvement or complete resolution following BEST treatment. However, skin hyperpigmentation after BEST therapy (Fig. 4) was observed in 6 patients (Table 2).

**Table 2:** Comparison of symptom prevalence before and after BEST therapy.

Symptom	Cases before BEST	Cases after BEST
Swelling	12	8
Skin lesions	19	11
Livid discoloration	15	8
Redness	6	3
Superficial dysplastic veins	4	1
Hyperpigmentation	2	6
Blistering	1	0
Ulcerations	1	0
Cutaneous induration	1	1
Functional limitations (e.g., limited range of motion of joints)	10	3
Thrombophlebitis	3	0
Sensory abnormalities	2	1

The table summarizes the number of cases reported for each symptom prior to treatment and at follow-up after therapy, highlighting improvements across multiple clinical parameters.

### Complications and aftercare

No periinterventional complications were recorded in any of the patients. Allergic reactions or pulmonary complications were not reported neither periprocedural nor during follow-up. 5 patients developed postprocedural issues requiring additional therapy but without long-term sequelae (CIRSE grade 3 complications^11^): 3 patients developed localized inflammation at the treatment site following the BEST session, which resolved with anti-inflammatory and antibiotic therapy. One patient experienced a temporary restriction of knee motion after undergoing BEST treatment of a thigh malformation, which fully resolved within 8 weeks of physiotherapy. The patients had been informed about these potential complications prior to therapy. Additionally, a patient with a combined venous-lymphatic malformation of the right thigh reported lymphorrhea lasting 3 weeks after the second treatment session, which spontaneously resolved without further sequelae.

## Discussion

Our study demonstrates that BEST is an effective treatment for slow-flow vascular malformations in pediatric patients, leading to significant volume reduction and symptom improvement. Most patients experienced a decrease in lesion size after treatment, and the majority reported either complete symptom resolution or improvement. No cases of symptom worsening were observed, and complications were limited to transient, manageable side effects. The results of our prospective study align well with the findings reported in several published retrospective studies:

Schmidt et al.^12^ conducted a retrospective comprehensive multicenter study evaluating both patient-reported outcomes and clinical improvements following BEST treatment, demonstrating significant benefits, particularly in younger patients. Our study adds to the understanding of BEST by incorporating MRI-based volumetry as an additional measure of treatment success. By including volumetric analysis alongside clinical outcomes, we provide further insight into the effects of BEST, complementing the patient-reported experiences assessed in previous research.

Our findings align with those of Kostusiak et al.^9^, who demonstrated the effectiveness of Bleomycin Electrosclerotherapy (BEST) in vascular malformations. While their study included only patients with limited or no response to standard sclerotherapy, our cohort encompassed all pediatric patients undergoing BEST. Furthermore, 5 of our patients had received no therapy before BEST. Despite these differences, both studies observed a high rate of symptom improvement or resolution, reinforcing the efficacy of BEST. Kostusiak et al. suggested that BEST may reduce the number of treatment sessions needed compared to standard sclerotherapy, which is consistent with our finding that most patients required only one or two BEST sessions.

Our findings demonstrate that not only malformations resistant to these prior treatments can respond favorably to BEST, suggesting that this therapy option should not be limited to difficult-to-treat cases. We observed that patients with large-volume lesions seemed to benefit from multiple BEST sessions. While individual responses to therapy varied, repeated treatments appeared to progressively reduce the size and symptom severity of malformations, indicating the potential cumulative benefit of BEST over time. This suggests that for patients with substantial lesion burdens, a longer treatment course may be advantageous to maximize therapeutic outcomes.

One particular case of a combined veno-lymphatic malformation in the right leg highlighted the variable nature of response to BEST: In this instance, no significant volume reduction was observed after the first session. However, partial reduction was achieved following the second and third treatments. This raises an important consideration regarding the interaction between lesion growth and treatment effect. We suppose that in pediatric patients, where malformations are known to increase proportionally in size in conjunction with the child’s growth, the initial treatment may be outpaced by the lesion’s natural progression. Subsequent treatments, therefore, may be required to achieve significant improvement as the therapy catches up with lesion expansion. These findings underscore the importance of considering both lesion size and patient age when determining the treatment strategy for slow-flow malformations. The dynamic nature of lesion growth in pediatric patients suggests that a tailored approach, including multiple sessions of BEST therapy, may be necessary to achieve optimal outcomes.

Skin hyperpigmentation at the treatment site occurred in 6 of 24 patients (25%). Hyperpigmentation was observed both in areas where the malformation was already visible at the skin surface and at the insertion sites of the electrodes. Transient postprocedural effects requiring additional therapy occurred in 5 of 24 patients (20%). In the study by Schmidt et al.^12^ patient-reported skin hyperpigmentation was notably higher at 69%. They also documented minor and major complications in approximately 10% of BEST sessions, including rare but severe adverse events such as arterial spasms, persistent nerve injury, and disseminated intravascular coagulation. In comparison, our study has observed a higher complication rate within in a smaller patient cohort; however, no major adverse events occurred in our patients. This difference may be attributed to variations in patient age, lesion characteristics, follow-up duration, or methodological differences, such as questionnaire-based self-reporting versus follow-up assessments conducted in a clinical setting.

Bleomycin’s cytotoxicity raises concerns about dose-dependent side effects, particularly pulmonary fibrosis, which becomes more likely at higher cumulative doses.^13^ BEST requires lower doses than traditional bleomycin sclerotherapy without electroporation, potentially further reducing the risk of pulmonary complications. This lower dose appears to provide an effective treatment while maintaining a safer toxicity threshold, which is particularly important for pediatric patients who may be more susceptible to long-term side effects.

This study has both strengths and limitations. As a monocentric study, it may be subject to selection bias, as all patients were treated at a single center with specialized expertise. Furthermore, since our institution no longer performs bleomycin sclerotherapy without electroporation, we were unable to collect comparative data between BEST and non-BEST sclerotherapy. To increase generalizability, future studies should consider a multicenter design to evaluate the effectiveness of BEST across a broader range of clinical settings. Additionally, we experienced some loss to follow-up (6 of 30 patients), partly due to the COVID-19 pandemic and related travel restrictions, which impacted especially our international patients seeking specialized care. However, the prospective study design is a notable strength, allowing us to systematically track therapeutic responses as well as monitor for potential complications over a mean 25-month follow-up period, providing further insights into the safety and efficacy of this treatment.

## Conclusion

This study demonstrates the safety and clinical benefit of Bleomycin Electrosclerotherapy (BEST) for managing slow-flow vascular malformations in pediatric patients. In addition to clinical improvement, MRI volumetric analysis showed a measurable reduction in lesion size, further supporting the therapeutic effectiveness of BEST. The findings underscore the need for individualized treatment strategies and highlight the potential for future research to explore the indications and possible long-term benefits of a multi-session treatment approach to optimize therapeutic protocols.

## Data Availability

The datasets generated and/or analyzed during the current study are available upon reasonable request from the corresponding author.
